# Supplementary material for: The mitochondrial copper chaperone COX11 has an additional role in cellular redox homeostasis
Source: PLoS One. 2021 Dec 17;16(12):e0261465. doi: 10.1371/journal.pone.0261465 (PMC8682889; doi:10.1371/journal.pone.0261465)
Supplement: S1 Raw images — (PDF) [file pone.0261465.s001.pdf]

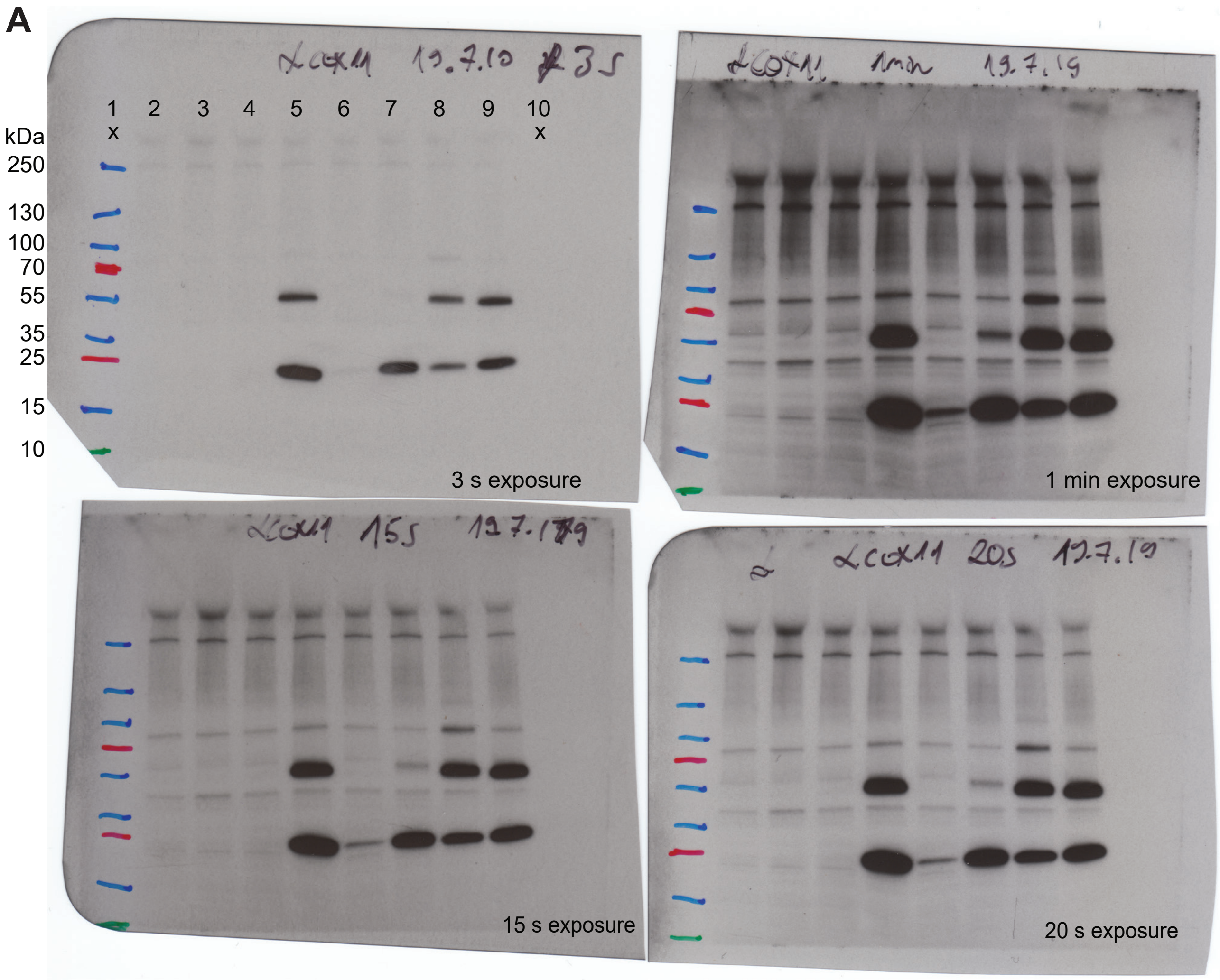

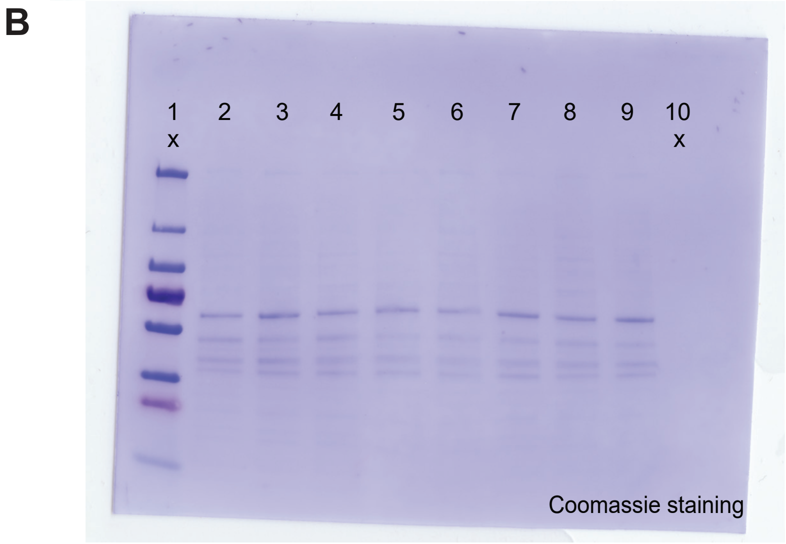

Loading order:
1. PageRuler plus prestained protein ladder (Thermo Scientific)
2. Empty vector
3. GFP
4. tAtCOX11
5. tScCOX11
6. tScCOX11 ΔCys
7. tScCOX11 C111A
8. tScCOX11 C208A
9. tScCOX11 C210A
10. Empty lane

S1 Raw Images

(**A**) Original scans of the Western blot X-ray films (developed in Protec Ecomax X-ray film processor). Lanes 2 to 9 contained 25 µg of total protein extracts from WT yeast transformed with a vector expressing different truncated COX11 variants. The 20 s exposure film was used in Fig. 4B. (**B**) Original scan of Coomassie staining of the same blot detected in A. For the Fig. 4B, the contrast was adjusted to make the protein bands easer to visualize. x, lanes not included in the Fig. 4B.
