# Supplementary material for: The mitochondrial copper chaperone COX11 has an additional role in cellular redox homeostasis
Source: PLoS One. 2021 Dec 17;16(12):e0261465. doi: 10.1371/journal.pone.0261465 (PMC8682889; doi:10.1371/journal.pone.0261465)
Supplement: S1 Fig — (PDF) [file pone.0261465.s002.pdf]

***AtCOX11**prom*

CATGTTGTAACAATGTTTTGGGCTTAGA**CTTGTG**TATATAAAAAG**ATGATCAA**ACTTATGGAATGT
ATATTAGTGAAGTTATTTGTGGAAGATTATTACAGAGAGATCCATCACAATAAATGAAATAATTTCG
TAATAACATCGGAGATATGGAAATCTTAAATGACAGCAGATTT**GTCCAAAA**TTAGATACATGTAAT
AA**ACACGTT**AAATTTAAGTATGAAATGACAATATTCCTTGTATACTCTAATTTTCATTTGGGCTTTA
TTGGGCCAAA**TAATATGG**GCTTACAATTAAAAATTCGACTGAACGGATGTGAATAATTTTT**TTTTG**
AAAGAACCGATTGATCTATCTTTATTCTTACCAGACCACAAAGACAAGTCGTTG**TTTGACT*TGTTG***
ACTTTTTTTTTAATC**CACAAG**TTTTTTTTTTTTTGTTGCGTCAGATTTTTTGTTTGTTTT**CCAGTG**AC
GTCTTTGATCTCCCTCCC**GCCGAC**GA**CACAAG**CACGCCGTCTGGTTTACCTGTAAGTCTTCTATT
CTCTTCTTTTCCTTT**CGCGT**GCTTCCTCTTTGATAGGTTACATTCTTTAATCGATCATGGCATCGTT
CTTGGCCTCTGATTCGATTACGCTCGGGTGATTTTTTTTTTTTCCCCCGATTATTGATCTCGCCGG
AGGTTGCGGAAGGGAG**AAATTGGG**TT**TCAAATTT**TC**GAAATTT**TCAAGTAATGTAATCTGGGTTC
GTGTTTATTGTGCAGATAAGTGTTTTTGTGTGGTGTCTGTGTCTCACTGTGTCGATAGATTAAAAT
AATTTCTCTTCGGCC**ATG...**

***AtHCC1**prom*

TG**AGTCAA**CTGGGTCTTCATGAAAAGAGTTGGAACTAAGTGATAGTAA**TGTTAAAA**CTGGGGGTG
GAAAGCAT**AAAT**ACTGAACGGCAAGGTGTTTGTATAAAGGGCGTTCGCAAACCTTGCCGTTTTAA
TATAAACTGTG**ATCT**AAAACAATTGATTGAAACCGGCTTAATGGGCTTT**AAATATCT**AAAACTAAT
GAGCTTGTCGACCCAAACGGGCTTTAAAAATAATTTATCAAATAAACAGAGAAGAAGAACCAAATT
CACTAGAAGGTTTTATAAAGTGTAAAACCTCTTTGCACCTCTCAGATTAAAAACCTCAGCTCGCAG
GAAAGAAATCAGCGAATTTGTGAACTTCAGAGAAAA**ATTGGTC**GTCA**ATG**....

***AtHCC2**prom*

CGGAATAACGGCGAGAAGGAAATGTGTGGTGGTGGAGGGAGTGTCGAGAGAGAAGAGAAGAGA
GACA**ACGCG**GAAATCAGGGTTTTTGCATGGAGTGGCAG**CAAGTGGC**TGTGGTTGATTTGCGTAA
CTTCAGTTGGTCTCTTTCGGACCGGAATTTTCTTTTGTTTACCAACTCAAATGTTTTTTTCTTCTTTT
CATGTCCCTCATTATTAGTATCAATTTTTTTTCTTTCTCATTTCCTAT**TTTGATCA**ATATAATTGTAA
ATTTTATATAGTCGCAAGTGAGGACTAAAATGATGGT**TTTGACT**AGGAACGATGGATAAATCACAC
TACTCACCCAACAAGAAAACATAATCTCATTGCAGATTTTCGACACAAAATATACTCAAATTGTTG
AATTAAAGCCCAATAAAACTCAAATTGTTGTATATAAAGCTCAATAATTTTTTGTGTGTCAATTATG
AAGCCCAATAATTTATTAGTAAGTCAACCCATAGATAATTTAATTACTTTACACAAAATTAAACAAA
TAAAAACATTAAAA**CACACG**GAAATGAGCTTGGCCACCAAGAAAAAAACA**AAAACTTT**TTTTATTT
TG**TTTTGGTT**TTGGCTCTTCACCAAGAAAAAAAGATTCTATAGTACGGAATAAGTTTTCTTTTGGAT
GAAACTGAAATAATTTATCAAATAAACAGAGAAGAAGAACCAAATTCA**ATG...**

S1 Fig. Cis-acting putative ROS-response elements in the putative promoter regions of *AtCOX11, AtHCC1,* and *AtHCC2*.

Based on overrepresentation in promoter regions of genes induced by singlet oxygen or other types of ROS, certain DNA sequences were published as ROS-responsive elements (ROSE). We found such ROSE in the promoter (prom) regions of AtCOX11, AtHCC1 and AtHCC2. Sequences in yellow were described by [Wang et al., 2013]. Superoxide- (in blue) and general ROS- (in green) specific motifs were identified by [Petrov et al., 2012]. Some motifs match more than one consensus sequence. Start codons are labelled with red letters. The 5’ untranslated regions (5’ UTR) are labelled in grey.
