# Supplementary material for: The mitochondrial copper chaperone COX11 has an additional role in cellular redox homeostasis
Source: PLoS One. 2021 Dec 17;16(12):e0261465. doi: 10.1371/journal.pone.0261465 (PMC8682889; doi:10.1371/journal.pone.0261465)
Supplement: S2 Fig — Total cell fluorescence intensity distributions of ScCOX11 knock-out (A, B, C) and overexpressing yeast (D, E, F) cells stained with DCFDA. (PDF) [file pone.0261465.s003.pdf]

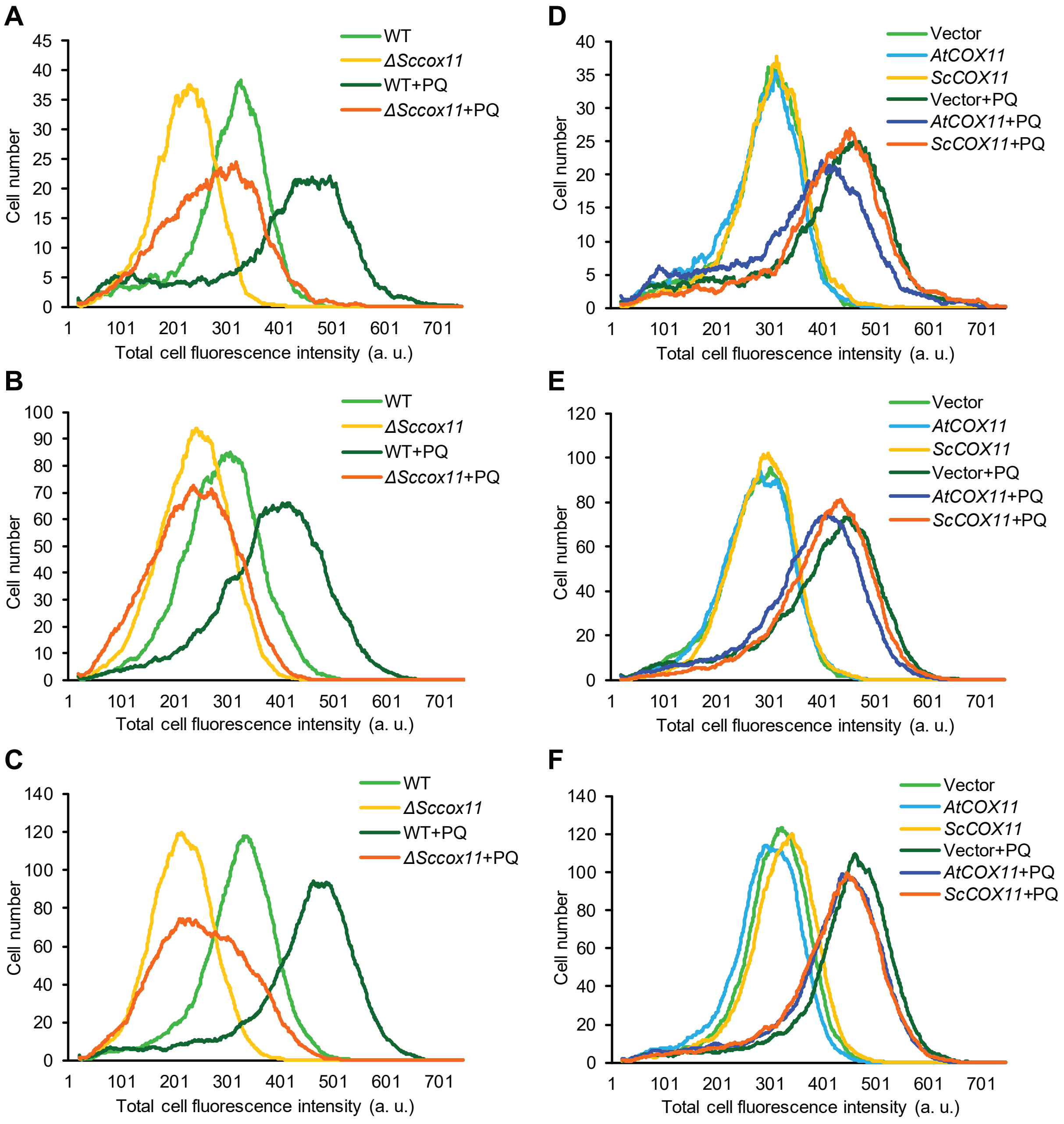

S2 Fig. Total cell fluorescence intensity distributions of *ScCOX11* knock-out (A, B, C) and overexpressing yeast (D, E, F) cells stained with DCFDA.

WT, *ΔSccox11* and *AtCOX11* or *ScCOX11* overexpressing yeast cells were either mock treated or treated with 2 mM paraquat (PQ) and stained with DCFDA to determine the cellular ROS levels. Total fluorescence of individual cells was measured by flow cytometry. Descriptive statistics for the cytometry datasets is given in **S1 Table**. **(A,D)(B,E)(C,F)** Data derived from three independent experiments. To reduce noise, a moving average (of 10 a. u.) was used to generate graphs which are, for clarity, depicted as lines. Vector = WT strain transformed with the empty vector used for overexpression. a. u. = arbitrary unit.
