# Supplementary figures and images for: The mitochondrial copper chaperone COX11 has an additional role in cellular redox homeostasis
Source: PLoS One. 2021 Dec 17;16(12):e0261465. doi: 10.1371/journal.pone.0261465 (PMC8682889; doi:10.1371/journal.pone.0261465)

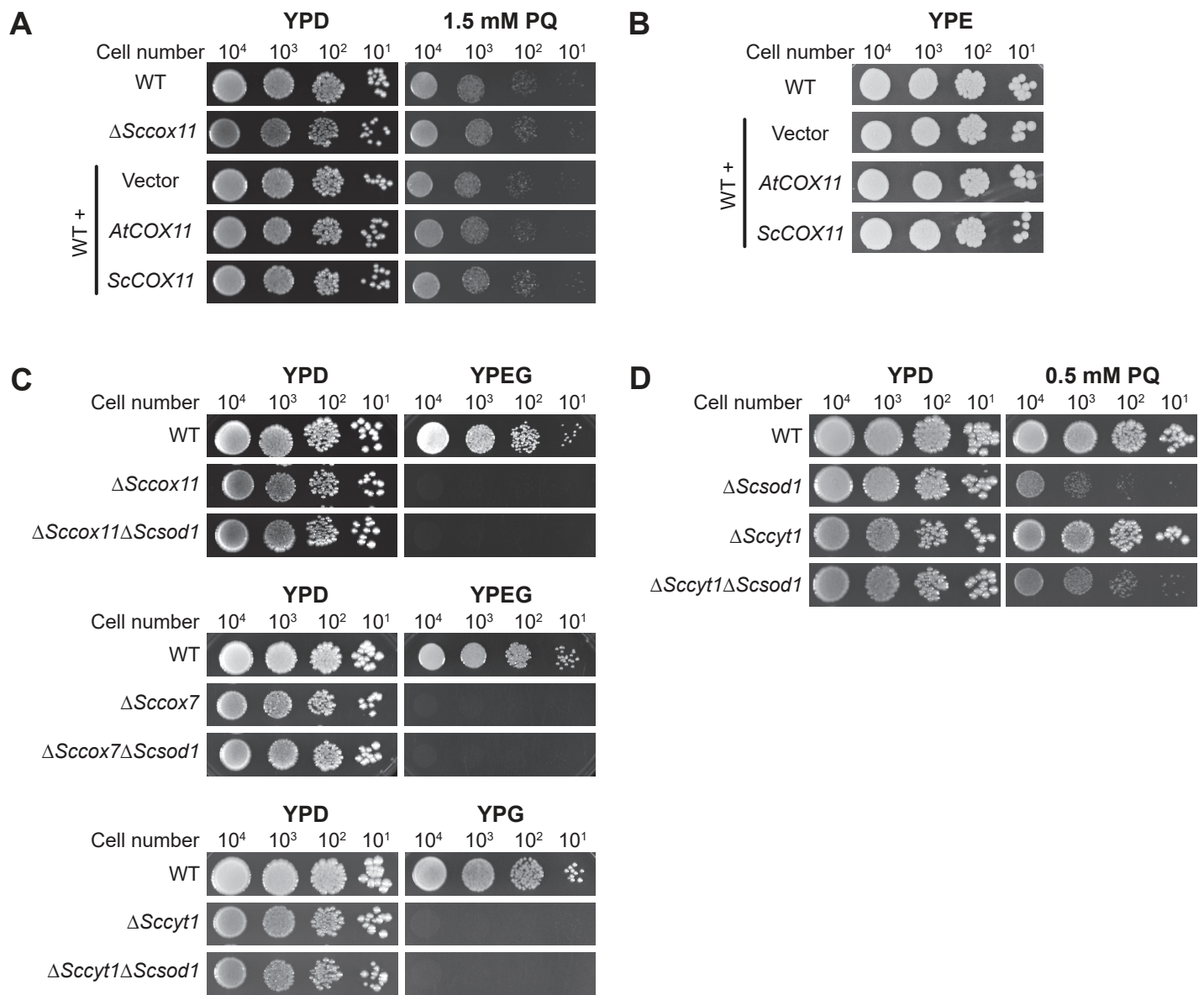

Supplement: S3 Fig — (PDF) [file pone.0261465.s004.pdf]
