# Supplementary material for: The mitochondrial copper chaperone COX11 has an additional role in cellular redox homeostasis
Source: PLoS One. 2021 Dec 17;16(12):e0261465. doi: 10.1371/journal.pone.0261465 (PMC8682889; doi:10.1371/journal.pone.0261465)
Supplement: S2 Table — (PDF) [file pone.0261465.s007.pdf]

**S2 Table.** Gene regulation in response to 50 µM antimycin A (microarray data from Ng et al. [2013], Genevestigator).

| Gene name | *AtCOX11* | *AtHCC1* | *AtHCC2* | *AtCOX5b-1* | *AtCRK21* | *AtAOX1a* |
|---|---|---|---|---|---|---|
| Fold change | +2.23 | +1.03 | +1.60 | -2.14 | -1.25 | +9.51 |

Arabidopsis plants were treated for 3 h with either mock treatment or with 50 µM antimycin A. GEO repository number: GSE36011.
