# Supplementary material for: The mitochondrial copper chaperone COX11 has an additional role in cellular redox homeostasis
Source: PLoS One. 2021 Dec 17;16(12):e0261465. doi: 10.1371/journal.pone.0261465 (PMC8682889; doi:10.1371/journal.pone.0261465)
Supplement: S3 Table — (PDF) [file pone.0261465.s008.pdf]

S3 Table. Cloning primers

#	Name	Sequence (5’ → 3’)
1	tAtCOX11 F	GGGGACAAGTTTGTACAAAAAAGCAGGCTACCATGTACGCTGCTGTGCCATTG
2	tScCOX11 F	GGGGACAAGTTTGTACAAAAAAGCAGGCTACCATGTATGCAGCGGTACCACTC
3	tAtCOX11 C119A F	TACGCTGCTGTGCCATTGTATAGAACATTCGCCCAAGC
4	tScCOX11 C111A F	TATGCAGCGGTACCACTCTATAGAGCCATTGCTGCTCG
5	AtCox11 R	GGGGACCACTTTGTACAAGAAAGCTGGGTTTAATTGGTTTCTTGAACTGG
6	ScCox11 R	GGGGACCACTTTGTACAAGAAAGCTGGGTTTAATTTGAGTTGTCTTTCC
7	tAtCOX11 C221A OL-F	CAACAAGATACAATGTTTTGCCTTTGAGGAGCAGCGACTC
8	tAtCOX11 C221A OL-R	GAGTCGCTGCTCCTCAAAGGCAAAACATTGTATCTTGTTG
9	tScCOX11 C210A OL-F	CCAATGTTTTGCCTTTGAAGAACAAAAGCTAGCTGCC
10	tScCOX11 C210A OL-R	GGCAGCTAGCTTTTGTTCTTCAAAGGCAAAACATTGG
11	tAtCOX11 TOPO F	CACCATGTACGCTGCTGTGCCATTG
12	tAtCOX11 TOPO R	TTAATTGGTTTCTTGAACTGGAAC
13	tScCOX11 TOPO F	CACCATGTATGCAGCGGTACCACTC
14	tScCOX11 TOPO R	TTAATTTGAGTTGTCTTTCCTTGTG
15	tAtCOX11 C219A OL-F	CAAGATACAAGCTTTTTGCTTTG
16	tAtCOX11 C219A OL-R	CAAAGCAAAAAGCTTGTATCTTG
17	tScCOX11 C208A OL-F	TAAAATCCAAGCTTTTTGCTTTG
18	tScCOX11 C208A OL-R	CAAAGCAAAAAGCTTGGATTTTA
19	tAtCOX11 Δcys OL-F	CAAGATACAAGCTTTTGCCTTTGAGGAGC
20	tAtCOX11 Δcys OL-R	GCTCCTCAAAGGCAAAAGCTTGTATCTTG
21	tScCOX11 Δcys OL-F	AATAAAATCCAAGCTTTTGCCTTTGAAGAAC
22	tScCOX11 Δcys OL-R	GTTCTTCAAAGGCAAAAGCTTGGATTTTATT
23	GFP-F	GGGGACAAGTTTGTACAAAAAAGCAGGCTACCATGGTGAGCAAGGGCGAGGAG

24	GFP-R	GGGGACCACTTTGTACAAGAAAGCTGGGTTTACTTGTACAGCTCGTCCATG

- tAtCOX11 was made by PCR with primers #1/5, followed by BP reaction into pDONR221-P1-P2.
- tScCOX11 was made by PCR with primers #2/6, followed by BP reaction into pDONR221-P1-P2.
- tAtCOX11 C119A was made by primer extension PCR with primers #1/3/5, followed by BP reaction into pDONR221-P1-P2.
- tScCOX11 C111A was made by primer extension PCR with primers #2/4/6, followed by BP reaction into pDONR221-P1-P2.
- tAtCOX11 C221A was made by overlap PCR with primers #1/7/8/5, followed by BP reaction into pDONR221-P1-P2.
- tScCOX11 C210A was made by overlap PCR with primers #1/9/10/5, followed by BP reaction into pDONR221-P1-P2.
- tAtCOX11 C219A was made by overlap PCR with primers #11/12/15/16, followed by D-TOPO reaction into pENTR.
- tScCOX11 C208A was made by overlap PCR with primers #13/14/17/18, followed by D-TOPO reaction into pENTR.
- tAtCOX11 Δcys was made by overlap PCR with primers #11/12/19/20 with tAtCOX11 C119A as template, followed by D-TOPO reaction into pENTR.
- tScCOX11 Δcys was made by overlap PCR with primers #13/14/21/22 with tScCOX11 C111A as template, followed by D-TOPO reaction into pENTR.
- GFP was made by PCR with primers #23/24 followed by BP reaction into pDONR221-P1-P2.
