# Supplementary material for: The mitochondrial copper chaperone COX11 has an additional role in cellular redox homeostasis
Source: PLoS One. 2021 Dec 17;16(12):e0261465. doi: 10.1371/journal.pone.0261465 (PMC8682889; doi:10.1371/journal.pone.0261465)
Supplement: S4 Fig — (PDF) [file pone.0261465.s005.pdf]

```
A.t.  MSWSKACRGTRISSYLENLHRTSQYPRTILCSRYYTHGACKSNEHYLRSK  50
                                        ....|.    .:|||
S.c.  -----------------------------------MIRICP----IVRSK  11

A.t.  RVFWGS---SSSWSLNSHSATAKSML--DSAHRQYSTHS--PSET-----  88
      ....|:   |.|| |..|:...:..:  :.|.|.||.:|  |..|
S.c.  VPLLGTFLRSDSW-LAPHALALRRAICKNVALRSYSVNSEQPKHTFDISK  60

A.t.  --------------------KSQKMLYYLTAVVFGMVGLTYAAVPLYRTF 118
                          |.:.:.:|.::|....:||.||||||||..
S.c.  LTRNEIQQLRELKRARERKFKDRTVAFYFSSVAVLFLGLAYAAVPLYRAI 110

A.t.  CQATGYGG---TVQRKETVEEKIARHSESGTVTEREIVVQFNADVADGMQ 165
      |..||:||   |.:||.|.::.|.      ..||:.|.:.|.::|:..:.
S.c.  CARTGFGGIPITDRRKFTDDKLIP------VDTEKRIRISFTSEVSQILP 154

A.t.  WKFTPTQREVRVKPGESALAFYTAENKSSAPITGVSTYNVTPMKAGVYFN 215
      |||.|.||||.|.|||:|||||.|:|.|...|.|::||::.|.:|..|||
S.c.  WKFVPQQREVYVLPGETALAFYKAKNYSDKDIIGMATYSIAPGEAAQYFN 204

A.t.  KIQCFCFEEQRLLPGEQIDMPVFFYIDPEFETDPRMDGINNLILSYTFFK 265
      ||||||||||:|..||:|||||||:|||:|.:||.|..|:::||.||||:
S.c.  KIQCFCFEEQKLAAGEEIDMPVFFFIDPDFASDPAMRNIDDIILHYTFFR 254

A.t.  VSEENTTETVNNNNSVPVQETN----------------------------  287
      ....:.| .|:::...|   |.|
S.c.  AHYGDGT-AVSDSKKEP—-EMNADEKAASLANAAILSPEVIDTRKDNSN  300
```

S4 Fig. Alignment of *A. thaliana* (*A. t.*) and *S. cerevisiae* (*S. c.*) COX11 protein sequences.

Sequences were retrieved from the GenBank database and aligned with the EMBOSS needle tool from the European Bioinformatics Institute. Vertical lines (I) indicate identical residues between the two sequences, while colons (:) and periods (.) represent conservative substitutions with strongly and weakly similar properties, respectively. Cysteines are labelled yellow, only last three of which are retained in the mature protein. The putative copper-binding motifs are boxed. The predicted cleavable N-terminal targeting signals are double underlined. The predicted transmembrane domains are shown in bold red letters. The bold black vertical line indicates the beginning of the truncated versions of the COX11 proteins (tCOX11).
