# Supplementary material for: The mitochondrial copper chaperone COX11 has an additional role in cellular redox homeostasis
Source: PLoS One. 2021 Dec 17;16(12):e0261465. doi: 10.1371/journal.pone.0261465 (PMC8682889; doi:10.1371/journal.pone.0261465)
Supplement: S4 Table — (PDF) [file pone.0261465.s009.pdf]

S4 Table. Primers used for qPCR.

Locus	Primer Name	Primer Sequence (5' → 3')	Conc. each (µM)	Efficiency (%)	Amplicon Size (bp)		Comment	Reference
					cDNA	gDNA		
At1g02410	*COX11* F<br>*COX11* R	GATTGACATGCCGGTCTTCT<br>TGGTTTCTTGAACTGGAACAGA	0.33	102.6	164	N/A	spans exon/exon	(Radin et al., 2015)
At1g13320	*PP2A* F<br>*PP2A* R	CCTGCGGTAATAACTGCATCT<br>CTTCACTTAGCTCCACCAAGCA	0.33	95.5	142	356	spans 2 introns	(Czechowski et al., 2005)
At3g18780	*ACT2* F<br>*ACT2* R	GGTAACATTGTGCTCAGTGGTGG<br>AACGACCTTAATCTTCATGCTGC	0.33	100.5	108	108	spans no introns	(Czechowski et al., 2005)
At3g22370	*AOX1a* F<br>*AOX1a* R	GGAGGCTTCCTGCTGATGCGACA<br>AGCTGGAGCTTCCTTTAGTTCACGACC	0.16	102.7	134	214	spans 1 intron	(Radin et al., 2015)
At3g08950	*HCC1* F<br>*HCC1* R	GGCCCGATCTTACCGGGTTT<br>CAACGCCGTCTGTCAACGAG	0.25	99.8	158	391	spans 1 intron	(Radin et al., 2015)
At4g23290	*CRK21 F*<br>*CRK21 R*	TCGTCCAAGCATGTCCACAA<br>CTCTCGGCTAATGGGTTCGG	0.5	101.1	120	120	spans no introns	This work
At3g15640	*COX5b-1* F<br>*COX5b-1* R	GACAAGCGAATTGTGGGCTG<br>AGTACTGAGTGCAAACCGGG	0.25	104.3	112	238	spans 1 intron	(Radin et al., 2015)
At4g39740	*HCC2 F*<br>*HCC2 R*	CGGATGTTGGACCTGAGCA<br>TTGCACTTGCAGTCCCGGTTA	0.25	97.0	189	379	spans 2 introns	This work

The optimal final primer concentrations (conc.) and primer pair efficiencies were experimentally determined. The amplicon size of the cDNA and of the potential contamination genomic DNA (gDNA) are given. F = forward, R = reverse, N/A = not applicable.

Czechowski T, Stitt M, Altmann T, Udvardi MK, Scheible WRR. Genome-wide identification and testing of superior reference genes for transcript normalization in *Arabidopsis*. Plant Physioliology. 2005;139: 5-17. doi: 10.1104/pp.105.063743

Radin I, Mansilla N, Rödel G, Steinebrunner I. The Arabidopsis COX11 Homolog is Essential for Cytochrome *c* Oxidase Activity. Frontier in Plant Science. 2015;6:1091. doi: 10.3389/fpls.2015.01091
