# Supplementary material for: The mitochondrial copper chaperone COX11 has an additional role in cellular redox homeostasis
Source: PLoS One. 2021 Dec 17;16(12):e0261465. doi: 10.1371/journal.pone.0261465 (PMC8682889; doi:10.1371/journal.pone.0261465)
Supplement: Attachment — Submitted filename: Response to reviewers_2021.docx [file pone.0261465.s010.docx]

**Comments to the Author**

*We thank all the reviewers for their comments and suggestions, which helped us improve this manuscript.*

1. Is the manuscript technically sound, and do the data support the conclusions?

The manuscript must describe a technically sound piece of scientific research with data that supports the conclusions. Experiments must have been conducted rigorously, with appropriate controls, replication, and sample sizes. The conclusions must be drawn appropriately based on the data presented.

Reviewer #1: No

Reviewer #2: Yes

Reviewer #3: Partly

Reviewer #4: Yes

2. Has the statistical analysis been performed appropriately and rigorously?

Reviewer #1: Yes

Reviewer #2: Yes

Reviewer #3: Yes

Reviewer #4: Yes

3. Have the authors made all data underlying the findings in their manuscript fully available?

The [PLOS Data policy](http://www.plosone.org/static/policies.action#sharing) requires authors to make all data underlying the findings described in their manuscript fully available without restriction, with rare exception (please refer to the Data Availability Statement in the manuscript PDF file). The data should be provided as part of the manuscript or its supporting information, or deposited to a public repository. For example, in addition to summary statistics, the data points behind means, medians and variance measures should be available. If there are restrictions on publicly sharing data—e.g. participant privacy or use of data from a third party—those must be specified.

Reviewer #1: Yes

Reviewer #2: Yes

Reviewer #3: Yes

Reviewer #4: Yes

4. Is the manuscript presented in an intelligible fashion and written in standard English?

PLOS ONE does not copyedit accepted manuscripts, so the language in submitted articles must be clear, correct, and unambiguous. Any typographical or grammatical errors should be corrected at revision, so please note any specific errors here.

Reviewer #1: Yes

Reviewer #2: Yes

Reviewer #3: Yes

Reviewer #4: Yes

5. Review Comments to the Author

Please use the space provided to explain your answers to the questions above. You may also include additional comments for the author, including concerns about dual publication, research ethics, or publication ethics. (Please upload your review as an attachment if it exceeds 20,000 characters)

**Reviewer #1:** This study focuses on COX11, a copper chaperone that delivers Cu++ to the cytochrome c oxidase. The authors propose that Cox 11 has an independent role in defense against reactive oxygen species. This was based on the observations that (1) COX11 mRNA is upregulated in Arabidopsis under oxidative stress, albeit COX11 knockdown lines have reduced ROS levels; (2) yeast strains overexpressing COX11 have lower levels of ROS; (3) the yeast cox11 sod1 double mutant is more sensitive to oxidative stress than the single mutant controls; (4) expression of COX11 in the cytosol enhances resistance to oxidative stress; (5) several cysteine residues are required for increased resistance to oxidative stress.

Although the hypothesis is appealing, the manuscript has numerous weaknesses. First, most phenotypes described are very marginal.

*Some of the observed differences are small. However, we believe that is to be expected as COX11 performs its physiological role in a very small compartment of mitochondrial intermembrane space. Other larger compartments likely mask changes within the intermembrane space. We discuss this point in the updated manuscript.*

The lack of a direct correlation between COX11 level and ROS production does not come up with a strong conclusion supporting a role of COX11 in scavenging ROS. The effect of overexpression may be indirectly related to ROS detoxification.

*We have modified the manuscript to reflect this valid concern. We conclude that COX11 is involved in cellular oxidative homeostasis and only discuss ROS scavenging ability as one possible explanation.*

Increased sensitivity of the sod1 cox11 double mutant to PQ is not unexpected, which can be interpreted as increased electron leak out of the membrane and not necessarily loss of defense mechanism.

*We have added a new control to address this concern (Fig. 3C, S3C). We show that the cox7sod1 (COX7 is a structural COX subunit) double mutant does not have the increased sensitivity to oxidative stress as observed for the cox11sod1 double mutant. This control shows that increased sensitivity of cox11sod1 mutant is unlikely to be a consequence of loss of COX complex activity and other related effects.*

The ectopic expression of Cox11 in the cytosol is not physiologically relevant. It cannot be excluded that the protein is toxic and affects menadione uptake.

*We rewrote the discussion to include these possibilities.*

Loss of cysteine may affect protein stability.

*We performed Western blots and found that single cysteine mutants were in fact stable. Only deletion of all three cysteines led to increased instability (Fig. 4B). We modified our conclusions to reflect that finding.*

**Reviewer #2:** Cox 11 is a copper chaperone for the maturation of cytochrome C oxidase. It has been suggested that it also plays a role in cellular defense against oxidants. In this manuscript, the authors provided further evidence that Cox 11 proteins in plants and the budding yeast play a role in cellular redox homeostasis. Two important results of this manuscript are: a cox11 mutation exacerbates the oxidant sensitivity phenotype of sod1 mutant cells; overexpression of three serine to alanine mutant alleles of cox11, all of which have been reported to result in respiratory deficiency, have differential effects in protecting cells against oxidants. The paper is technically sound and well written. I have three concerns, which, hopefully, can be addressed.
1. cox11 and sod1 mutant cells should be slow growing due to a loss of respiratory function and oxidative stress, respectively. In figure 3C, strains grown on YPD medium showed comparable growth. Could this be due to growth saturation on plate after several days? Would you be able to see noticeable growth differences among the strains after one or two days growth on YPD plate?

*We have added growth curve experiments performed in liquid media to address this (Fig 3C and S3C). We found that most of the mutants grow only marginally slower compared with the WT on YPD media. This is likely because YPD is so rich. In our experience, respiratory deficient stains show only minimal growth retardation in the YPD medium.*

2. The authors should express COX11 from a plasmid in sod1 cox11 double mutant cells and show that the growth defect on YPD + PQ plate is now like that of sod1 mutant cells.

*We have performed that experiment and observed a partial phenotype rescue. However, we opted not to include those results in the manuscripts as they were quite variable. This was likely due to a combination of inherent variabilities both in ROS growth assay and transient protein expression.*

3. It is a bit strange to me that the COX11 alleles without the mitochondrial targeting signal and the transmembrane domain are referred to as being soluble. Should authors use cytosolic expression of Cox11 without the mitochondrial targeting signal and the transmembrane domain instead?

*We modified the text to be more accurate in our descriptions.*

**Reviewer #3:** The manuscript by Radin and colleagues investigates possible role of Complex IV assembly factor COX11 in ROS protection in plant Arabidopsis thaliana and Saccharomyces cerevisiae. The role of Cox11 in assembly of Complex IV of mitochondrial respiratory chain was well characterized in yeast and bacteria, where its essential function is to delivery copper for copper b site (CuB) of subunit COX1 in Complex IV. In addition to this role Veniamin et al. (2011) reported increased sensitivity to hydrogen peroxide of ScCox11 null mutants which suggested additional function of this protein. Here the authors focused on model organism A. thaliana. In previous publication the same group reported role of COX11 in Complex IV assembly (similar to yeast and bacteria) also in A. thaliana, where the plant development was negatively effected in knock-down of COX11 (KD) in A. thaliana. In this manuscript the authors used as a model mutated plants from previous research (knock-down KD and overexpressing OE versions of COX11) and speculate about another role of COX11 in oxidative stress protection. We can separate the study into two parts – in the first one the authors investigate secondary role of COX11 in A. thaliana and in the second part in S. cerevisiae.
In A. thaliana the authors show that AtCOX11 mRNA levels are upreagulated upon oxidative stress, using representative controls (positive and negative) as well as another Complex IV assembly factor AtHCC1 which does not behave consistently upon different treatment. Next they measure ROS cellular levels in mutant plants (KD and OE). Here the only difference is in KD plants where the ROS levels are reduced. This is more likely effect of respiratory chain dysfunction due to KD of COX11 as essential factor for Complex IV assembly. Because lower ROS levels can be observed also in other mutants of Complex IV assembly factors in different organisms.
In the second part the authors use S. cerevisiae Cox11 null mutant and wild type (WT) overexpressing ScCOX11 and AtCOX11. In the previous publication authors showed that AtCOX11 does not complement COX11 function in S. cerevisiae. Here upon oxidative stress treatment the ROS levels are lower in WT + AtCOX11 / ScCOX11 vs. WT suggesting that COX11 protective function has to be independent of the copper delivery function for Complex IV. The manuscript brings new possible function of COX11.
Specific issues:
1) Crucial experiment is to show protective function of COX11 in A. thaliana. I understand that handling with A. thaliana is more challenging than to work with yeasts. Still the authors should try similar assay as they used in yeast – treated WT and OE plants with oxidative stressor to show increase of ROS in WT and significantly smaller increase of ROS in OE plants. Or explain why they can not perform the assay or why it is not working.

*While we do agree that these experiments would be interesting, it is indeed technically very challenging to perform oxidative stress tests in plants. A major complication for ROS studies in plants is the presence of chloroplasts which generate huge amounts of ROS and chloroplast mitochondrial cross-talk through photorespiration. It would take a considerable time to optimize the assay, find the appropriate growth stage and tissue, etc. Unfortunately, the authors with the plant expertise have now moved on to different positions making this process difficult. In addition, even if we would successfully perform the experiment, we believe it wouldn’t bring any new substantial conclusion to the manuscript. Our main conclusions are based on our yeast experiments which we have expanded in this updated manuscript.*

2) The authors should also elucidate why the plants OE COX11 has developmental issues. In this manuscript it is suggested that COX11 has protective function. How this inconsistency can be explained?

*We discuss the very minor developmental issues of OE COX11 plants in our previous publication where these lines were generated (Radin et al,* *Frontiers in Plant Science 2015). There we discussed the possibility that overexpression of COX11 leads to disruption of copper delivery in mitochondria; COX11 binds too much copper, limiting access for HCC1, which affects proper COX assembly. Indeed, in that manuscript, we were able to show that COX11 OE plants have lower COX activity and likely lower energy supply, which would affect plant organs dependent on respiration like roots, the most.*

3) Using S. cerevisiae model to be able to perform more difficult experiments, the authors should also demonstrate in yeast model that ScCOX11 mRNA levels are upregulated upon oxidative stress including controls.

*Two publicly available microarrays on oxidatively stressed yeast cells did not find a significant change in ScCOX11 expression, at least under experimental conditions and time points the authors used. It is possible that under different conditions change could be observed. Regardless, this does not affect the conclusions from our yeast experiments presented here, which are based on cellular responses.*

4) PQ requires membrane potential and working respiratory chain for import into mitochondria. It is possible that this function can be compromised in S. cerevisiae overexpressing AtCOX11 or ScCOX11 due to too high steady state level of COX11 which can also negatively effect the mitochondria. As good control the authors could perform western blot analysis of WT and OE yeasts and plants to show steady-state levels of Complex IV (as COX1, COX2, COX11 or COX19). Or by some other methods prove that the mitochondria in OE strains are working properly.

*We performed and added a control experiment to address this (S3B Fig). We cultured WT and OE yeast on YPE (ethanol) media and showed that their growth is indistinguishable suggesting that OE stains have normal respiration.*

5) How the authors can explain results from Fig.3 A and C. Where PQ in WT cells increase ROS level (Fig. 3A) but the growth of the cells is not effected (Fig. 3C). How significant in that case can be the function of COX11 in the cell?

*Data from Fig. 3A and C (in the previous version of the manuscript) are hard to compare directly. In Fig., 3A yeast is exposed to acute stress with high levels of PQ (2 mM) while in Fig 3C the stress is prolonged and at a lower level (0.2 mM PQ) meaning that cells have more to adjust. In the discussion, we argue that COX11 likely doesn’t serve as a primary defense but possibly as an additional mechanism, e.g., when sod1 fails and or under high ROS levels.*

6) To show better resolution of PQ effect on S. cerevisiae growth in Fig. 3C, the authors should perform growth curve instead of growing the cells on the YPD plates. ΔScCox11 looks like it grows a little bit less in PQ than WT.

*We have performed and added this experiment (Fig. 3C and S3C).*

7) In Fig. 4B, the menadione treatment is very lethal for WT cells. As control for the protective function the authors could try to use plates with 9 mM hydrogen peroxide (like in Martínez-Garay, Carlos Andrés et al., 2016, Applied and environmental microbiology). In addition the authors should perform also negative control – to overexpress at least one of the genes which do not respond consistently upon oxidative treatment such as ScSCO1 and AtHCC1 or ScSCO2 and AtHCC2, which should not have protective function.

*The menadione concentration was chosen to be lethal for the WT, so only cells that overexpress COX11 are able to grow, which suggests that COX11 helps alleviate some of the stress. We included a negative control (GFP), which was chosen because of its similar size to COX11 and lack of any known antioxidative activity. Sco1, Sco2, and their Arabidopsis homologs could potentially convey some level of protection to cells, similarly to COX11 would not be optimal negative controls.*

8) To complete Fig. 4B authors could perform western blot analysis of the overexpressing forms of Cox11 and demonstrate that all the proteins are same stabile overexpress and the difference between rescues by different forms of Cox11 is not caused due to different levels of the proteins in the cell.

*We have performed and added this experiment (Fig. 4B), and updated our discussion and conclusions to reflect the new data.*

9) Despite overexpression of AtCOX11 in ΔScCox11 null mutant does not rescue the phenotype of the cells; in this study the authors show no difference between AtCOX11 and ScCox11 in stress response protection in yeasts. This can be explained only as these two functions of COX11 are not connected. In the discussion the authors can speculate about possible cause.

*We discuss this now.*

**Reviewer #4:** The intermembrane space of mitochondria is a small cellular compartment of outstanding physiological relevance. It contains many proteins that contain disulfide bonds, either for structural or for catalytic reasons. Cox11 and Sco1/Sco2 are intermembrane space enzymes that are tethered to the inner membrane. Both factors contain conserved cysteine residues and play a direct or indirect role in the metalation of cytochrome oxidase. Sco1/Sco2 was proposed to carry out two functions, one in assembly of Cox2 and one as a thioredoxin-like redox enzyme. The situation for Cox11 is less clear. It is required for copper insertion into Cox1 but its catalytic, biochemical activity is unknown.
In the present study the authors propose that Cox11 plays a role in the “defence against oxidative stress”. They show evidence based on experiments with Arabidopsis and yeast Cox11. Mutants with reduced Cox11 levels appear to have higher levels of “ROS”. Yeast strains, in which the catalytic domain of Cox11 was artificially overexpressed in the cytosol, show an increased resistance against menadione.
This is an interesting and technically sound study on a conserved protein of high physiological relevance. It remains unclear whether under physiological conditions, the function of Cox11 for redox resistance is independent of its role in cytochrome oxidase biogenesis. Nevertheless, this study will help to better understand the role of Cox11 and inspire more detailed analyses on this important factors. Some minor aspects should be considered:

1. Title: “Oxidative Stress”: There is little evidence for a specific role of Cox11 to counteract oxidative conditions. The evidence is: (i) In plants, expression of Cox11 is upregulated upon oxidative stress conditions – but this is presumably true for many other genes as well. (ii) Overexpression of Cox11 in the cytosol provides menadione resistance. The experiment shown here on overexpressed soluble Cox11 is very artificial and does not tell much about the physiological function of Cox11. I therefore suggest to change the title slightly and write: “The mitochondrial copper chaperone COX11 plays a role in cellular redox homeostasis”

*Thank you for the suggestions, we have amended the title.*

2. The way how “ROS” was measured here is not really informative. The concept of ROS-mediated oxidative stress in cells is misleading and outdated as H2O2 is an important signaling molecule. It would be important to measure the levels of H2O2, superoxide or GSSG in different cellular compartments to elucidate its role in redox homeostasis. There are targeted molecular sensors available to measure these parameters in yeast. However, this will go beyond the scope of this study. Nevertheless, the authors should be more specific about the changes described with the MDA, HAE and DCF measurements. What does this exactly mean? Maybe they discuss their results with a redox biologist.

*We agree that measurements of MDA, HAE, and DCF are not easy to interpret, as they are products of complex pathways. Nevertheless, here we focus on the involvement of COX11 in oxidative homeostasis and so we use ROS levels as an umbrella term that covers multiple types of molecules with different characteristics and metabolism. Undoubtedly in the future, it would be interesting to parse apart the complex networks of ROS signaling. We also clarified our wording in the manuscript.*

3. There is no doubt that Cox11 is crucial for cytochrome oxidase biogenesis. Therefore, data from COX-deficient strains need to be shown for comparison in Fig. 2 and Fig. 3.

*We have added COX deficient strains to our yeast experiments (Fig. 3C, S3C).*

4. The levels of soluble Cox11 in the strains need to be controlled by Western blotting. From the data shown it is unclear whether the cysteine-to-alanine mutants are not functional or not stable. Data obtained with polyclonal Cox11 antibodies were published before and such sera are presumably available in the yeast community.

*We have performed and added this experiment (Fig. 4B). And updated our discussion and conclusions to reflect the new data.*

5. The authors claim that the redox function of Cox11 is independent from its role in COX biogenesis. To support this claim, the authors need to show that expression of soluble Cox11 also provides menadione resistance in a COX-deficient (Δcox4) or mtDNA-deficient strain background.

*Our soluble (now named truncated) COX11 variants do not have mitochondria targeting signals, so they are unable to affect the COX function. This limitation of targeting uncouples the two COX11 roles.*

6. If you would like your identity to be revealed to the authors, please include your name here (optional).

Your name and review will not be published with the manuscript.

Reviewer #1: (No Response)

Reviewer #2: (No Response)

Reviewer #3: (No Response)

Reviewer #4: Johannes Herrmann
